# Dangerous Delicacy: Contaminated Sea Turtle Eggs Pose a Potential Health Threat

**DOI:** 10.1289/ehp.117-a407b

**Published:** 2009-09

**Authors:** John Tibbetts

**Affiliations:** **John Tibbetts**, based in Charleston, South Carolina, has written for *EHP* since 1995. Editor of *Coastal Heritage*, the magazine of the South Carolina Sea Grant Consortium, he is a member of the Society of Environmental Journalists

The eggs of the green turtle (*Chelonia mydas*) and other sea turtle species are a popular food in areas such as Peninsular Malaysia—so popular, in fact, that nesting populations in the region have declined by more than 80% since the 1950s, largely because of their eggs being collected for human consumption. Persistent organic pollutants (POPs) and heavy metals have been reported in the eggs of a number of *C. mydas* populations. Now a team of Australian and Malaysian scientists reports that the concentrations of POPs found in *C. mydas* eggs from markets in Peninsular Malaysia could pose a considerable threat to human health **[*EHP*** **117:1397–1401; van de Merwe et al.]**.

In August 2006, the investigators surveyed 33 markets along 730 miles of coastal Peninsular Malaysia. *C. mydas* eggs were available in 9 of these 33 markets. A random sample of 3–13 eggs was purchased from each market where they were sold. In total, 55 eggs were collected and frozen until they could be analyzed.

The eggs were analyzed for numerous POPs, among them 83 polychlorinated biphenyls (PCBs), 23 organochlorine pesticides, and 19 polybrominated diphenyl ethers. Eggs were also analyzed for zinc, copper, cobalt, selenium, arsenic, cadmium, lead, and mercury. For each metal and category of POP, the authors calculated the percentage of the acceptable daily intake (ADI) found in the eggs, providing an estimate of potential human health risks involved in consuming the eggs. ADIs are set by the World Health Organization.

The concentrations of POPs and metals measured were generally lower than those reported elsewhere for loggerhead sea turtle (*C. caretta*) eggs. Nevertheless, all the eggs analyzed had at least 3 times the ADI of coplanar PCBs, which are among the most toxic members of their chemical family. One egg had 300 times the ADI for this contaminant.

The authors note that the rate of *C. mydas* egg consumption in Peninsular Malaysia was not investigated in the present study, nor has it been well quantified. However, there is a cultural perception in this area that sea turtle eggs have medicinal qualities. The authors write that a public education campaign could highlight the health consequences of consuming contaminated eggs. This in turn could reduce egg collection pressure and hence potentially contribute to the recovery of the *C. mydas* populations in this region.

## Figures and Tables

**Figure:**
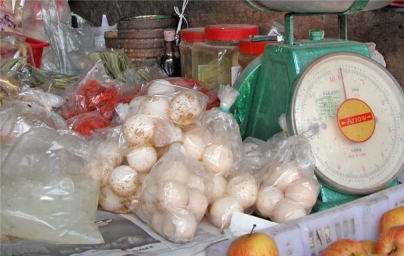
*C. mydas* eggs for sale in Kuala Terengganu, Penisular Malaysia

